# Severe Hypokalemia and Life-Threatening Ventricular Arrhythmias: A Case of Normotensive Primary Aldosteronism

**DOI:** 10.7759/cureus.109525

**Published:** 2026-05-23

**Authors:** Hilary N Daniel, Vicki Belleus, Jeffrey Wang, Seth Adjovu

**Affiliations:** 1 Internal Medicine, Dignity Health Saint Rose Dominican Hospital, Las Vegas, USA

**Keywords:** aldosterone-to-renin ratio (arr), captopril suppression test, chronic hypokalemia, conn's syndrome, primary hyperaldosteronism, ventricular arrthymias

## Abstract

Primary aldosteronism (PA), the most common cause of secondary hypertension, is characterized by autonomous adrenal aldosterone overproduction independent of the renin-angiotensin system, resulting in sodium retention, potassium wasting, and cardiovascular sequelae that exceed the risk attributable to blood pressure elevation alone. Although hypertension is the predominant clinical manifestation, PA may also present without hypertension or hypokalemia, making atypical presentations a significant diagnostic challenge. Aldosterone-mediated myocardial fibrosis combined with hypokalemia-driven electrophysiologic instability can precipitate life-threatening ventricular arrhythmias that may constitute the dominant clinical presentation in the absence of classic features. We present a case of normotensive PA in which diagnostic delay resulted in recurrent ventricular arrhythmias and sudden cardiac arrest before identification of the underlying etiology, underscoring the importance of aldosterone-to-renin ratio (ARR) screening in patients with unexplained treatment-resistant hypokalemia and arrhythmia regardless of blood pressure status. Early recognition and targeted treatment of PA in atypical presentations are essential to mitigate irreversible cardiovascular morbidity.

## Introduction

Primary aldosteronism (PA) is a disorder of renin-independent aldosterone excess and is increasingly recognized as a major contributor to secondary hypertension and cardiovascular morbidity. Aldosterone-mediated activation of renal mineralocorticoid receptors and increased expression of epithelial sodium channels (ENaCs) result in increased sodium reabsorption and potassium excretion [1]. This leads to sodium retention, volume expansion, and suppression of renin secretion, manifesting as hypertension in most affected individuals and hypokalemia in a subset [1,2]. Although hypokalemia has historically been regarded as a prominent feature of PA, recent data indicate that it is present in only 9-37% of affected individuals, with normokalemic hypertension constituting the predominant clinical presentation [3]. 

Less commonly recognized is normotensive PA, which has been reported in approximately 10% of individuals with renin-independent hyperaldosteronism [4]. PA is associated with cardiovascular risk that exceeds that attributable to the degree of blood pressure elevation alone. Aldosterone exerts direct pathological effects on the cardiovascular system, including promotion of left ventricular hypertrophy and myocardial interstitial and perivascular fibrosis, which may serve as the mechanistic basis for arrhythmogenesis, as well as endothelial dysfunction, which may further contribute to cardiovascular risk [4]. The association between severe hypokalemia and ventricular arrhythmias is well established through multiple mechanisms involving altered Na⁺/K⁺-ATPase activity, intracellular calcium overload, prolongation of the action potential duration, and generation of triggered afterdepolarizations [5]. Pooled analyses of observational data demonstrate that individuals with PA experience substantially higher rates of atrial fibrillation, stroke, myocardial infarction, and heart failure than those with essential hypertension at similar blood pressure levels [4].

We present the case of a normotensive patient who experienced recurrent ventricular arrhythmias, sudden cardiac arrest, and severe treatment-resistant hypokalemia, for which the unifying etiology remained unidentified over a prolonged clinical course. Prior management included catheter ablation and implantation of an implantable cardioverter-defibrillator (ICD). The subsequent identification of PA as the causative etiology in this normotensive individual underscores the importance of maintaining a high index of suspicion for this condition even in the absence of hypertension, particularly when persistent hypokalemia refractory to conventional supplementation is a dominant clinical feature [6].

## Case presentation

A 42-year-old woman with a history of atrial and ventricular septal defects surgically repaired in infancy presented to the outpatient primary care clinic with persistent fatigue and progressive weight gain. Her medical history was notable for a sudden cardiac arrest in 2021, four years before presentation, presumed secondary to ventricular arrhythmias in the setting of severe hypokalemia. Following this event, she underwent catheter ablation and implantation of an ICD and was maintained on long-term oral potassium supplementation since 2021. Despite supplementation, serum potassium levels remained persistently below the normal range, prompting further evaluation for secondary causes of hypokalemia. Measurement of plasma aldosterone concentration (PAC) to plasma renin activity (PRA) ratio revealed a markedly elevated aldosterone-to-renin ratio (ARR) of 90 ng/dL/hr (reference range: <15 ng/dL/ng/mL/hr) [1], findings suggestive of primary aldosteronism (Table 1).

**Table 1:** Patient's aldosterone level, plasma renin level and aldosterone-to-renin ratio suggesting primary hyperaldosteronism PA - primary aldosteronism; LC/MS - liquid chromatography-tandem mass spectrometry The criteria for diagnosing primary aldosteronism were based on the Endocrine Society guidelines [1].

Parameter	Patient Value	Unit	Meets criteria for PA
Serum aldosterone LC/MS	9	ng/dL	≥ 7.5 ng/dL
Plasma renin activity (PRA)	0.10	ng/mL/h	≤ 1 ng/mL/h
Aldosterone-to-renin ratio (ARR)	90	ng/dL/ng/mL/hr	> 15 ng/dL/ng/mL/hr

To evaluate for an aldosterone-producing adrenal lesion, noncontrast computed tomography (CT) of the abdomen was performed and revealed a 1.6 × 1.1 cm hypodense nodule in the right adrenal gland with an attenuation of 5 Hounsfield units (HU) (Figure 1). Given the clinical and biochemical findings suggestive of PA, empiric therapy with the mineralocorticoid receptor antagonist was initiated with spironolactone 25 mg daily, and the patient was urgently referred to endocrinology and interventional radiology for adrenal venous sampling and consideration of surgical management. However, the patient subsequently relocated abroad, and care was transferred to her country of origin for further evaluation and definitive management.

**Figure 1:**
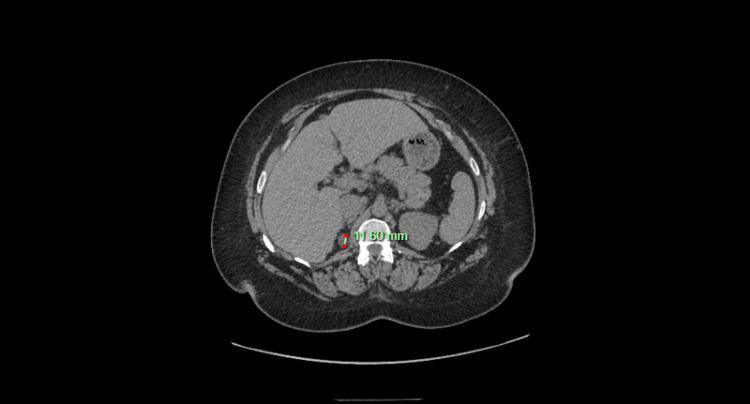
Patient's unenhanced axial computed tomography of the abdomen demonstrating a 1.4 × 1.1 cm hypodense right adrenal nodule

## Discussion

PA is one of the most common and, in cases of unilateral disease, potentially curable causes of secondary hypertension, with an estimated prevalence of 5 - 10% among hypertensive populations [3]. Importantly, PA warrants consideration even in normotensive patients or those with only mild blood pressure elevation, as the absence of overt hypertension does not exclude the diagnosis. This case illustrates the diagnostic challenges inherent in identifying PA in a patient presenting with an atypical clinical profile, including normotension, a complex congenital and acquired cardiac history, and chronic treatment-resistant hypokalemia without a previously identified cause.

The requirement for continuous potassium supplementation over a four-year period, despite the absence of identifiable gastrointestinal or renal losses, suggested persistent mineralocorticoid excess and raised clinical suspicion for aldosterone-mediated potassium wasting as a contributing factor to the patient's recurrent ventricular arrhythmias. Additionally, the patient's only medications were levothyroxine and potassium supplements, eliminating the possibility of medication interference in biochemical evaluation. Biochemical evaluation revealed an elevated ARR of 90 ng/dL per ng/mL/hr, with a suppressed PRA of 0.10 ng/mL/hr (reference range: 0.25 - 5.82 ng/mL/hr) and a PAC of 10 ng/dL (reference range: 3 - 28 ng/dL). Notably, the 2016 Endocrine Society Clinical Practice Guidelines recommend that ARR interpretation incorporate both the absolute PAC and the degree of renin suppression, as false-positive results may occur when aldosterone levels fall below the recommended screening threshold of 7.5-10 ng/dL in the setting of profoundly suppressed renin [1,3]. In this case, although the PAC was at the lower limits of the recommended threshold, the combination of suppressed renin, chronic treatment-resistant hypokalemia, a radiographically apparent unilateral adrenal lesion, and a history of life-threatening ventricular arrhythmias collectively supported the clinical suspicion for PA and warranted confirmatory testing.

Confirmatory testing was performed using the captopril suppression test (CST). Following administration of captopril, the PAC decreased from 10 ng/dL to 5 ng/dL, with a corresponding post-captopril ARR of 17.2 ng/dL per ng/mL/hr (Table 2). Both the post-captopril aldosterone level and ARR fell below commonly cited diagnostic thresholds for confirmation of PA, findings that would conventionally argue against autonomous aldosterone secretion [4]. However, several factors may have confounded the interpretation of these results. Hypokalemia is a well-established suppressor of aldosterone biosynthesis and may attenuate baseline aldosterone levels if not corrected before testing. In this clinical context, an artifactually low baseline PAC could exaggerate the apparent degree of post-captopril suppression, increasing the likelihood of a false-negative result [4,7]. Moreover, percentage-based suppression is less diagnostically reliable when the baseline aldosterone concentration is already near the lower screening threshold, as in this case. The Endocrine Society guidelines acknowledge that no single confirmatory test has perfect sensitivity and that clinical context must inform diagnostic decision-making [3,8]. Taken together, these considerations support interpreting the negative CST with caution in the context of this patient’s overall presentation.

**Table 2:** Patient's plasma aldosterone concentration, plasma renin activity, and aldosterone-to-renin ratio during captopril suppression testing LC/MS - liquid chromatography-tandem mass spectrometry

Parameter	Baseline (two hours pre-captopril)	Post-captopril (50mg)	Unit
Serum aldosterone LC/MS	10	5	ng/dL
Plasma renin activity (PRA)	0.28	0.29	ng/mL/h
Aldosterone-to-renin ratio (ARR)	35.7	17.2	ng/dL/ng/mL/hr

The CT findings of a unilateral hypodense adrenal nodule with low attenuation were radiographically consistent with a lipid-rich adrenal adenoma (Figure 1). However, imaging alone cannot confirm that a visualized nodule is the functional source of aldosterone excess, as nonfunctioning adenomas may coexist with bilateral adrenal hyperplasia [9]. Adrenal incidentalomas are common, with a reported prevalence of approximately 4-5% in radiographic series and up to 6% in autopsy studies, the majority of which are nonfunctioning cortical adenomas [10,11]. Furthermore, aldosterone secretion in PA, although autonomous from the renin-angiotensin system, remains subject to modulation by secondary stimuli, including adrenocorticotropic hormone (ACTH), gonadotropin-releasing hormone (GnRH), serotonin agonists, and upright posture [12]. This variability may produce fluctuations in aldosterone levels that transiently fall within the normal range, potentially yielding false-negative results on biochemical or confirmatory testing [12].

Beyond its effects on renal electrolyte handling, aldosterone acts on mineralocorticoid receptors expressed in nonepithelial tissues, including cardiomyocytes, vascular smooth muscle cells, and fibroblasts [4]. Activation of these receptors promotes myocardial inflammation, oxidative stress, and interstitial fibrosis, contributing to left ventricular hypertrophy, diastolic dysfunction, and arterial wall remodeling [4,13]. These structural changes are associated with an increased risk of adverse cardiovascular outcomes, including stroke, myocardial infarction, and heart failure [9]. Aldosterone excess has also been linked to QT interval prolongation, which, in combination with hypokalemia, may further amplify arrhythmogenic susceptibility [14]. In addition, PA is associated with a higher prevalence of metabolic syndrome, endothelial dysfunction, and microalbuminuria, reflecting the systemic scope of aldosterone-mediated end-organ injury [15].

Confirmatory testing may yield equivocal or negative results despite a clinical phenotype strongly suggestive of mineralocorticoid excess. No single confirmatory test has perfect sensitivity, and clinical context must guide diagnostic decision-making [9]. When diagnostic uncertainty persists and clinical suspicion remains high, empiric mineralocorticoid receptor antagonist therapy may be appropriate, as in this patient. Accordingly, the patient was referred to endocrinology for further evaluation, including consideration of adrenal venous sampling and determination of surgical candidacy.

The patient's history of surgically repaired atrial and ventricular septal defects represents an additional potential contributor to arrhythmia susceptibility through residual conduction abnormalities and myocardial scarring. However, the presence of structural heart disease should not deter investigation into coexisting metabolic causes of arrhythmias, including chronic treatment-resistant hypokalemia.

## Conclusions

This case highlights the limitations of applying rigid biochemical thresholds to a disorder characterized by secretory variability and susceptibility to confounding by concurrent electrolyte abnormalities. The diagnosis of PA should integrate biochemical, radiographic, and clinical data rather than rely on any single criterion, particularly in patients with significant cardiovascular morbidity, in whom a missed diagnosis may have serious consequences. Normotensive primary aldosteronism is an uncommon but clinically important presentation that may manifest with severe hypokalemia and life-threatening arrhythmias, including syncope and sudden cardiac arrest. Screening for hyperaldosteronism should therefore be considered in patients with chronic unexplained hypokalemia, regardless of blood pressure status.
